# Emergence of Drug Resistance Is Associated with an Increased Risk of Death among Patients First Starting HAART

**DOI:** 10.1371/journal.pmed.0030356

**Published:** 2006-09-19

**Authors:** Robert S Hogg, David R Bangsberg, Viviane D Lima, Chris Alexander, Simon Bonner, Benita Yip, Evan Wood, Winnie W. Y Dong, Julio S. G Montaner, P. Richard Harrigan

**Affiliations:** 1 British Columbia Centre for Excellence in HIV/AIDS, St. Paul's Hospital, Vancouver, British Columbia, Canada; 2 Department of Medicine, Faculty of Medicine, University of British Columbia, Vancouver, British Columbia, Canada; 3 Department of Health Care and Epidemiology, Faculty of Medicine, University of British Columbia, Vancouver, British Columbia, Canada; 4 Epidemiology and Prevention Interventions Center, Division of Infectious Diseases and the Positive Health Program, San Francisco General Hospital, San Francisco, California, United States of America; 5 Department of Pathology and Laboratory Medicine, Faculty of Medicine, University of British Columbia, Vancouver, British Columbia, Canada; National Institutes of Health, United States of America

## Abstract

**Background:**

The impact of the emergence of drug-resistance mutations on mortality is not well characterized in antiretroviral-naïve patients first starting highly active antiretroviral therapy (HAART). Patients may be able to sustain immunologic function with resistant virus, and there is limited evidence that reduced sensitivity to antiretrovirals leads to rapid disease progression or death. We undertook the present analysis to characterize the determinants of mortality in a prospective cohort study with a median of nearly 5 y of follow-up. The objective of this study was to determine the impact of the emergence of drug-resistance mutations on survival among persons initiating HAART.

**Methods and Findings:**

Participants were antiretroviral therapy naïve at entry and initiated triple combination antiretroviral therapy between August 1, 1996, and September 30, 1999. Marginal structural modeling was used to address potential confounding between time-dependent variables in the Cox proportional hazard regression models. In this analysis resistance to any class of drug was considered as a binary time-dependent exposure to the risk of death, controlling for the effect of other time-dependent confounders. We also considered each separate class of mutation as a binary time-dependent exposure, while controlling for the presence/absence of other mutations. A total of 207 deaths were identified among 1,138 participants over the follow-up period, with an all cause mortality rate of 18.2%. Among the 679 patients with HIV-drug-resistance genotyping done before initiating HAART, HIV-drug resistance to any class was observed in 53 (7.8%) of the patients. During follow-up, HIV-drug resistance to any class was observed in 302 (26.5%) participants. Emergence of any resistance was associated with mortality (hazard ratio: 1.75 [95% confidence interval: 1.27, 2.43]). When we considered each class of resistance separately, persons who exhibited resistance to non-nucleoside reverse transcriptase inhibitors had the highest risk: mortality rates were 3.02 times higher (95% confidence interval: 1.99, 4.57) for these patients than for those who did not exhibit this type of resistance.

**Conclusions**

We demonstrated that emergence of resistance to non-nucleoside reverse transcriptase inhibitors was associated with a greater risk of subsequent death than was emergence of protease inhibitor resistance. Future research is needed to identify the particular subpopulations of men and women at greatest risk and to elucidate the impact of resistance over a longer follow-up period.

## Abstract 2

Emergence of resistance to both non-nucleoside reverse transcriptase inhibitors and protease inhibitors was associated with a higher risk of subsequent death, but the risk was greater in patients with NNRTI-resistant HIV.

## Editors' Summary

**Background.:**

In the 1980s, infection with the human immunodeficiency virus (HIV) was effectively a death sentence. HIV causes AIDS (acquired immunodeficiency syndrome) by replicating inside immune system cells and destroying them, which leaves infected individuals unable to fight off other viruses and bacteria. The first antiretroviral drugs were developed quickly, but it soon became clear that single antiretrovirals only transiently suppress HIV infection. HIV mutates (accumulates random changes to its genetic material) very rapidly and, although most of these changes (or mutations) are bad for the virus, by chance some make it drug resistant. Highly active antiretroviral therapy (HAART), which was introduced in the mid-1990s, combines three or four antiretroviral drugs that act at different stages of the viral life cycle. For example, they inhibit the reverse transcriptase that the virus uses to replicate its genetic material, or the protease that is necessary to assemble new viruses. With HAART, the replication of any virus that develops resistance to one drug is inhibited by the other drugs in the mix. As a consequence, for many individuals with access to HAART, AIDS has become a chronic rather than a fatal disease. However, being on HAART requires patients to take several pills a day at specific times. In addition, the drugs in the HAART regimens often have side effects.

**Why Was This Study Done?:**

Drug resistance still develops even with HAART, often because patients don't stick to the complicated regimens. The detection of resistance to one drug is usually the prompt to change a patient's drug regimen to head off possible treatment failure. Although most patients treated with HAART live for many years, some still die from AIDS. We don't know much about how the emergence of drug-resistance mutations affects mortality in patients who are starting antiretroviral therapy for the first time. In this study, the researchers looked at how the emergence of drug resistance affected survival in a group of HIV/AIDS patients in British Columbia, Canada. Here, everyone with HIV/AIDS has access to free medical attention, HAART, and laboratory monitoring, and full details of all HAART recipients are entered into a central reporting system.

**What Did the Researchers Do and Find?:**

The researchers enrolled people who started antiretroviral therapy for the first time between August 1996 and September 1999 into the HAART Observational Medical Evaluation and Research (HOMER) cohort. They then excluded anyone who was infected with already drug-resistant HIV strains (based on the presence of drug-resistance mutations in viruses isolated from the patients) at the start of therapy. The remaining 1,138 patients were followed for an average of five years. All the patients received either two nucleoside reverse transcriptase inhibitors and a protease inhibitor, or two nucleoside and one non-nucleoside reverse transcriptase inhibitor (NNRTI). Nearly a fifth of the study participants died during the follow-up period. Most of these patients actually had drug-sensitive viruses, possibly because they had neglected taking their drugs to such an extent that there had been insufficient drug exposure to select for drug-resistant viruses. In a quarter of the patients, however, HIV strains resistant to one or more antiretroviral drugs emerged during the study (again judged by looking for mutations). Detailed statistical analyses indicated that the emergence of any drug resistance nearly doubled the risk of patients dying, and that people carrying viruses resistant to NNRTIs were three times as likely to die as those without resistance to this class of antiretroviral drug.

**What Do These Findings Mean?:**

These results provide new information about the emergence of drug-resistant HIV during HAART and possible effects on the long-term survival of patients. In particular, they suggest that clinicians should watch carefully for the emergence of resistance to NNRTIs in their patients. Because this type of resistance is often due to poor adherence to drug regimens, these results also suggest that increased efforts should be made to ensure that patients comply with the prescribed HAART regimens, especially those whose antiretroviral therapy includes NNRTIs. As with all studies in which a group of individuals who share a common characteristic are studied over time, it is possible that some other, unmeasured difference between the patients who died and those who didn't—rather than emerging drug resistance—is responsible for the observed differences in survival. Additional studies are needed to confirm the findings here, and to investigate whether specific subpopulations of patients are at particular risk of developing drug resistance and/or dying during HAART.

**Additional Information.:**

Please access these Web sites via the online version of this summary at http://dx.doi.org/10.1371/journal.pmed.0030356.

- US National Institute of Allergy and Infectious Diseases fact sheet on HIV infection and AIDS
- US Department of Health and Human Services information on AIDS, including details of approved drugs for the treatment of HIV infection
- US Centers for Disease Control and Prevention information on HIV/AIDS
- Aidsmap, information on HIV and AIDS provided by the charity NAM, which includes details on antiretroviral drugs

## Introduction

Post-surveillance cohort studies and long-term follow-up studies of clinical trials, like the Merck 035 study, continue to suggest the ongoing durability of highly active antiretroviral therapy (HAART) [1–3]. Mortality among persons on these three- or four-drug regimens continues to remain low, and may be no higher than for other chronic diseases [4,5]. As one large multinational study indicates, depending upon the baseline characteristics of the persons initiating HAART, subsequent disease progression and death can be as low as 2.4% at 3.5 y [4].

Emerging resistance poses a growing threat to the ongoing success and durability of HAART regimens. Nearly 80% of antiretroviral-treated patients with detectable viraemia in one large US-based clinic study had evidence of phenotypic drug resistance [6]. The frequency of resistance in this population-based study was significantly higher in patients with a history of antiretroviral use, advanced HIV disease, higher plasma HIV viral load, and the lowest CD4 cell counts. Continuing viral replication during therapy leads to the accumulation of drug-resistance mutations, resulting in increased viral load.

The current impact of the emergence of drug-resistance mutations on mortality, however, is not well characterized in antiretroviral-naïve patients first starting HAART. Patients may have sustained immunologic function with resistant virus [7], and there is limited evidence that reduced sensitivity to antiretrovirals leads to rapid disease progression or death [8]. Previously, we documented the selection of resistance in an initially drug-naïve cohort starting HAART [9]. We therefore undertook the present analysis to characterize the determinants of mortality in this prospective cohort study with a median of nearly 5 y of follow-up. The aim of this study was to determine the impact of the emergence of drug-resistance mutations on survival among persons initiating HAART.

## Methods

### HIV/AIDS Drug Treatment Program

The distribution and the population-based monitoring of antiretroviral therapy in British Columbia have been extensively described in the literature [10]. Since 1986, 6,305 HIV-1-positive men and women have received antiretroviral therapy. Prescriptions are distributed through several designated pharmacies or through one of the 1,200 or more physicians who have ever prescribed antiretroviral therapy. Although the majority of HIV-1-positive men and women who have been given therapy are from Vancouver and the surrounding region, eligible treatment recipients have come from all regions in the province.

Since October 1992, the distribution of antiretrovirals has been the responsibility of the HIV/AIDS Drug Treatment Program of the British Columbia Centre for Excellence in HIV/AIDS (the Centre). The Centre distributes antiretroviral drugs based on guidelines generated by the Therapeutic Guidelines Committee, which is made up of physicians, pharmacists, virologists, health service researchers, and economists [11]. The Centre's HIV/AIDS Drug Treatment Program has received ethical approval from the University of British Columbia Ethics Review Committee at its St. Paul's Hospital site. The program also conforms with the province's Freedom of Information and Protection of Privacy Act.

### Data Collection

All antiretroviral treatment recipients in the province are entered into an Oracle-based monitoring and evaluation reporting system that uses standardized indicators to prospectively track the antiretroviral use and clinical and health status of HIV-1-positive individuals. Physicians enrolling an HIV-1-infected individual into the system must complete a drug request enrolment prescription form, which compiles information on the participant's address, past HIV-specific drug history, CD4 cell counts, plasma HIV-1 RNA, current drug requests, and enrolling physician data. Typically, persons receiving antiretroviral therapy are monitored by physicians at intervals no longer than 3 mo, at which time prescriptions are renewed or modified. At the time of the initial dispensation, participants are asked to provide informed consent for accessing medical electronic records (which may be used for health utilization studies but are not relevant to the analyses in this study) and to complete a participant survey, which elicits information on sociodemographic characteristics, clinical and health status, and alternative therapy use. Both the consent form and the participant survey are optional, and participant's refusal to do either does not limit his or her access to free antiretroviral therapy. At the same time, the treating physicians are asked to complete a clinical staging form using the World Health Organization clinical staging system.

The Centre recommends that plasma HIV-1 RNA levels and CD4 cell counts be monitored at baseline, at 4 wk after starting antiretroviral therapy, and every 3 mo thereafter. Plasma HIV-1 RNA levels were determined using the Roche Amplicor Monitor assay (Roche Diagnostics, Laval, Quebec, Canada) using either the standard method or the ultrasensitive adaptation. Plasma samples were stored chronologically as they were drawn and securely stored (frozen at −20 °C) for future use. CD4 cell counts were measured by flow cytometry, followed by fluorescent monoclonal antibody analysis (Beckman Coulter, Mississauga, Ontario, Canada).

Resistance testing was also completed on stored plasma HIV-1 RNA samples [12–14]. HIV RNA was extracted from plasma using the Qiagen (Huntsville, Alabama, United States) viral RNA kit using a BioRobot 9600/9604 or extracted manually using guanidinium-based buffer, followed by isopropanol and ethanol washes. Protease and reverse transcriptase genes were amplified from plasma HIV-1 RNA using nested RT-PCR as described previously [15]. PCR products were sequenced in both the 5′ and 3′ directions using an ABI 3700 or 3100 automated sequencer (Applied Biosystems, Foster City, California, United States), and a consensus sequence was generated. Results of the genotyping analysis are reported as amino acid changes in the HIV protease and reverse transcriptase sequences with respect to a wild-type reference sequence (HIV HXB2).

### Study Participants

All HIV-infected men and women in the current study were entered into the Centre's monitoring and evaluation system when they were first prescribed antiretroviral agents. Eligible study participants were persons who were antiretroviral naïve and were first dispensed triple combination therapy between August 1, 1996, and September 30, 1999. Participants must also have had a CD4 count and plasma HIV-1 RNA measurement within 6 mo of the first antiretroviral start date. Study data from eligible participants were extracted from the Centre's monitoring and evaluation system to form the HAART Observational Medical Evaluation and Research (HOMER) cohort.

For all HOMER cohort participants, HIV drug resistance genotyping was attempted on all samples with HIV-1 RNA levels of 1,000 copies/ml or more collected in the first 30 mo following initiation of HAART. HIV isolates were assigned to one of four resistance categories based on a modification of the International AIDS Society–USA table [11]. Samples were considered resistant if they displayed one or more major resistance mutations in one of four categories: lamivudine (184I/V), any other nucleoside reverse transcriptase inhibitors (41L, 62V, 65R, 67N, 69D or insertion, 70R, 74V, 75I, 151M, 210W, 215F/Y, or 219E/Q), any non-nucleoside reverse transcriptase inhibitors (100I, 103N, 106A/M, 108I, 181C/I, 188C/H/L, 190A/S, P225H, M230L, or 236L), and any protease inhibitors (30N, 46I/L, 48V, 50L/V, 54V/L/M, 82A/F/S/T, 84V, or 90M). Lamivudine resistance was analyzed as a separate category because of the very common appearance of this mutation and the lack of cross-resistance conferred to other nucleoside reverse transcriptase inhibitors. As genotyping does not yield consistently successful results on samples with low viral loads, samples with HIV-1 RNA levels of less than 1,000 copies/ml were not systematically genotyped and were assumed to have no drug resistance mutations.

### Outcome Measures and Explanatory Variables

The primary endpoint in this analysis was all cause mortality. Deaths occurring during the follow-up period were identified on a continuous basis from physician reports and through annual record linkages carried out with the British Columbia Division of Vital Statistics.

The following explanatory variables were investigated: age, gender, CD4 cell count, plasma HIV-1 RNA levels, prior AIDS diagnosis, protease inhibitor use, current or past history of injection drug use, physician experience, adherence, and drug resistance. Physician experience was defined as the number of HIV-positive patients the physician had previously treated at the time the study participant was enrolled into the HIV/AIDS Drug Treatment Program. Estimates of adherence to antiretroviral therapy are based on medications actually dispensed, not prescribed. Patients receive new prescriptions at various time intervals ranging from monthly to, at most, every 3 mo. For this exercise we limited our measure of adherence to the first year of therapy and estimated it by dividing the number of months of medications dispensed by the number of months of follow-up.

### Statistical Analyses

Cumulative mortality rates were estimated using Kaplan-Meier methods. Event-free participants were right censored as of June 30, 2003. Participants included in this analysis were not followed after this date, and those lost to follow-up were censored at the date of last known contact with the HIV/AIDS Drug Treatment Program.

Cox proportional hazard regression was used to model the simultaneous effect of prognostic variables on survival [16]. A forward stepwise technique was used in the selection of covariates. Interactions and non-proportionality of hazards were explored in our modeling. The assumption of proportional hazards was validated by inspection of log(−log[survival function]) estimates against log time plots.

In this analysis, a number of prognostic variables were treated as time-dependent variables including the following: protease inhibitor use, a diagnosis of AIDS, CD4 cell count, plasma HIV-1 RNA levels, and drug resistance. Protease inhibitor use (yes versus no), a diagnosis of AIDS (yes versus no), plasma HIV-1 RNA levels (<100,000 versus ≥100,000 copies/ml), emergence of resistance to antiretrovirals (yes versus no), and each class of resistance mutation (yes versus no) were treated as binary time-dependent variables. In our analysis we assumed that once resistance was detected, this variable was not allowed to change values until the end of the follow-up period. Other variables were measured at baseline and treated as either categorical or continuous. Gender (male versus female) and history of injection drug use (yes versus no) were treated as fixed binary variables. Age (in years) and physician experience (per 100 patients followed) were treated as continuous variables.

Marginal structural modeling was used to address potential confounding between time-dependent variables in the Cox proportional hazard regression models [17]. This potential confounding occurs when there exists a time-dependent covariate that is predictive of both mortality and the explanatory variable, but is also predicted by the explanatory variable itself. In this analysis, HIV-1 RNA is confounded with the exposure of interest, drug resistance. Marginal structural modeling is a form of causal analysis based upon counterfactuals, the hypothetical difference between the observed outcome for each individual and the unobserved outcomes that would have arisen had they received a different exposure history [18]. In essence, the model uses a series of logistic regression steps incorporating the time-dependent variables to weight the observations in such a way as to remove the confounding effects of the time-dependent variables while maintaining the original associations between the exposure variables and outcome. Unbiased estimates of the causal effect of exposure on the outcome can then be computed using the estimated weights for each observation and baseline predictors, provided that all important confounders have been considered and that the models used to compute the weights and the effects are properly specified [17]. HIV-1 RNA levels, CD4 cell count, AIDS diagnosis, and protease and non-nucleoside inhibitor use were considered as potential time-dependent confounders, while resistance to any class of drug (yes versus no) was considered as a time-dependent exposure. We also considered each separate class of mutation as a binary time-dependent exposure while controlling for the presence/absence of other mutations.

Analyses were performed using SAS software version 9.1.3 service pack 3 (SAS, Cary, North Carolina, United States). All tests of significance were two-sided, with a *p*-value of less than 0.05 indicating that an association was statistically significant.

## Results

Between August 1, 1996, and September 30, 1999, a total of 1,312 antiretroviral-naïve participants aged 18 y and over initiated triple combination therapy consisting of two nucleosides plus a protease inhibitor or a non-nucleoside reverse transcriptase inhibitor. Of these, 121 (9.2%) were excluded from this analysis for not having both baseline CD4 and plasma HIV-1 RNA level measures available within 6 mo prior to the start of antiretroviral therapy or for having initiated therapy as part of a clinical trial. Among the remaining 1,191 study participants, there were 679 patients with HIV-drug-resistance genotyping done on samples of plasma HIV-1 RNA before initiating HAART at the time of this analysis. HIV-drug resistance to any class was observed in 53 (7.8%) of the patients. Among these patients, 39 (73.6%) exhibited resistance to one class, ten (18.9%) to two classes, and four (7.6%) to three classes. A total of 14 (26.4%) exhibited mutations conferring resistance to lamivudine, 29 (54.7%) to other nucleoside reverse transcriptase inhibitors, eight (15.1%) to non-nucleoside reverse transcriptase inhibitors, and 19 (35.8%) to protease inhibitors. These patients exhibiting baseline resistance were excluded from the analysis, and the total study sample was based on the remaining 1,138 (86.7%) participants. Study participants had HIV-1 RNA levels higher than those of individuals excluded from the analysis (median viral load 87 versus 120 copies/ml; *p* < 0.001). No other significant associations were noted between the two groups.

Among the remaining HOMER participants (*n =* 1,138), a total of 229 (20.1%) patients achieved durable viral suppression (HIV-1 RNA levels < 1,000 copies/ml) during the entire follow-up period. There were 776 (68.2%) patients with at least one HIV-1 RNA level measurement at 1,000 copies/ml or above that showed no detectable resistance during the follow-up period, and we assumed these individuals had no drug resistance. Although we systematically genotyped all samples with HIV-1 RNA levels of 1,000 copies/ml or more during follow-up, there were three (0.3%) patients for whom the resistance test failed at least once for technical reasons, and for these particular tests we assumed that no mutation was detected.

Study participants were first prescribed 26 different triple combination antiretroviral regimens. Table 1 shows that over half of these participants (848; 74.5%) initiated therapy with a protease inhibitor, while the rest of the study participants (290; 25.5%) had a regimen that included a non-nucleoside reverse transcriptase inhibitor. A total of 123 (10.8%) commenced therapy in 1996, 402 (35.3%) in 1997, 340 (29.9%) in 1998, and 273 (24.0%) in 1999. The median age was 37 y (inter-quartile range: 32, 44 y), CD4 cell count was 280 cells/mm^3^ (inter-quartile range: 130, 430 cells/mm^3^), plasma HIV-1 RNA level was 120,000 copies/ml (inter-quartile range: 43,000, 310,500 copies/ml), physician experience was 44 patients per physician (inter-quartile range: 5, 131 patients per physician). There were 317 (27.9%) participants who had a history of injection drug use and 149 (13.1%) study participants with a prior diagnosis of AIDS. Among all participants, 956 were males (84.0%).

**Table 1:**
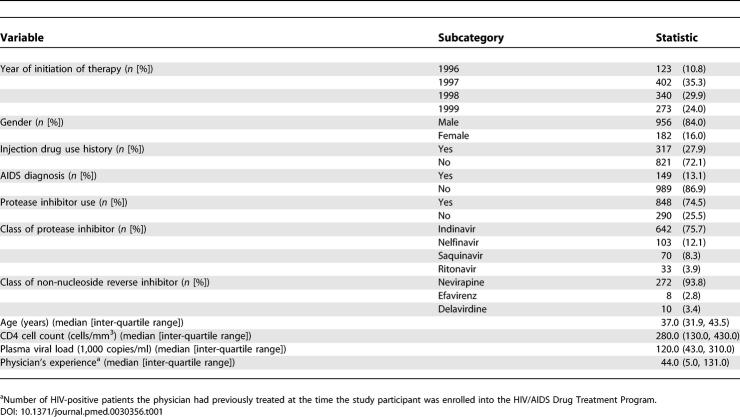
Baseline Characteristics of Patients Initiating Any Triple Combination Antiretroviral Therapy

The overall median time of follow-up was 56.4 mo (inter-quartile range: 45.8, 69.2 mo). The median number of CD4 count measurements was 3.59 per year (inter-quartile range: 2.24, 4.59 per year), and the median number of plasma HIV-1 RNA measurements was 3.44 per year (inter-quartile range: 2.56, 4.25 per year). A total of 17,632 CD4 cell counts and 17,358 HIV-1 RNA determinations were collected for participants over the study period. The median numbers of CD4 cell counts and HIV-1 RNA determinations per participant were 15 (inter-quartile range: 7, 23) and 15 (inter-quartile range: 9, 21), respectively. In this study, a switch in drug regimen was recorded when any modification (addition or removal) in the original drug regimen occurred. Men and women switched one or more drugs in their regimen a median three times (inter-quartile range: 1, 5 times) over the follow-up period. Protease inhibitors were added to or removed from a regimen a median of one time (inter-quartile range: 1, 3 times), non-nucleoside reverse transcriptase inhibitors one time (inter-quartile range: 0, 2 times), and nucleoside reverse transcriptase inhibitors three times (inter-quartile range: 1, 5 times). The median time to first switch was 7.8 mo (inter-quartile range: 2.1, 23.6 mo) in this study population.

Drug-resistance testing was completed on a total 3,099 samples over the first 30 mo of follow-up. A median of three (inter-quartile range: 1, 5) samples were tested per participant. HIV-drug resistance to any class was observed in 302 (28.5%) participants. Of these, 19 (6.3%) exhibited resistance to all three classes, 137 (45.4%) to two classes, and 146 (48.3%) to one class. A total of 221 (73.2%) exhibited mutations conferring resistance to lamivudine, 98 (32.5%) to other nucleoside reverse transcriptase inhibitors, 157 (52.0%) to non-nucleoside reverse transcriptase inhibitors, and 66 (21.9%) to protease inhibitors. The median time to first emergence of resistance to any class of drug under study was 16.5 mo (inter-quartile range: 8, 27 mo).

Table 2 presents the association between baseline characteristics, adherence during first yea,r and emergence of resistance. Persons who developed any mutations were more likely to be injection drug users (*p* = 0.007), to have lower CD4 counts (*p* < 0.001), and to have higher plasma viral load (*p* < 0.001) than persons who did not develop any resistance over the first 30 mo.

**Table 2:**
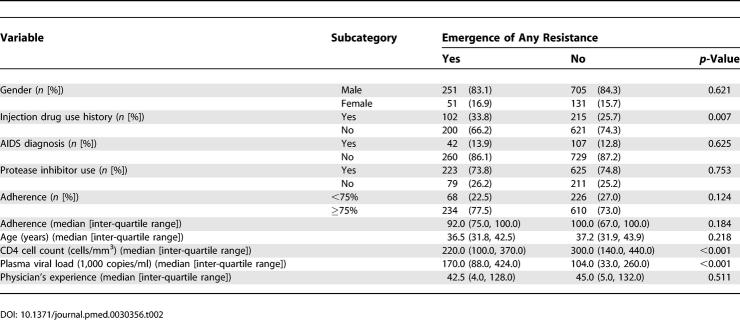
Associations between Baseline Variables and Emergence of Any Resistance in 1,138 Participants First Prescribed Any Triple Combination Antiretroviral Therapy

As of June 30, 2003, a total of 207 deaths were identified in the study population over the follow-up period, with an overall crude mortality rate of 18.2%. The product limit estimate of the cumulative mortality rate at 12 and 24 mo was 4.4% (±0.6%) and 8.7% (±0.8%), respectively. Of these deaths, 30 (14.5%) were to patients who exhibited resistance to one class, 26 (12.6%) to two classes, and one (0.5%) to three classes. A total of 41 (19.8%) deaths were to patients who exhibited resistance to lamivudine, 15 (7.3%) to other nucleosides, 32 (15.5%) to non-nucleoside reverse transcriptase inhibitors, and six (2.9%) to protease inhibitors. Nearly, three-quarters of the deaths involved no resistance to any class of drug (150; 72.5%).

The univariable and multivariable analyses of the baseline and time-dependent factors associated with all cause mortality are presented in Table 3 through four different models. In the univariable analysis for baseline characteristics (Model 1), only age, a prior AIDS diagnosis, protease inhibitor use, physician experience, CD4 count, and HIV-1 RNA level were associated with mortality. When we considered time-dependent factors, the univariable analysis (Model 2) showed us that age, baseline drug combination, physician experience, a prior AIDS diagnosis, protease inhibitor use, adherence, CD4 cell count, HIV-1 RNA level, and resistance to any antiretroviral were associated with mortality. We also observed that resistance to some classes of drug, more specifically to lamivudine and non-nucleoside reverse transcriptase inhibitors, were also associated with mortality. After controlling for other prognostic explanatory variables, Model 3 shows that there is a strong association between emergence of any resistance and risk of death (hazard ratio: 1.58 [95% confidence interval (CI): 1.14, 2.20]). The heterogeneity in the association between resistance and mortality also depends on the type of resistance that was acquired (Model 4), with persons who exhibited resistance to non-nucleoside reverse transcriptase inhibitors being 2.57 times (95% CI: 1.74, 3.78) more likely to die.

**Table 3:**
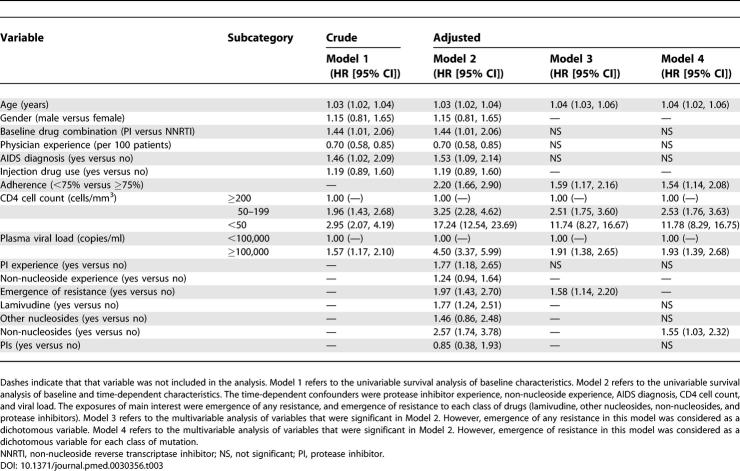
Univariable and Multivariable Analyses of the Baseline and Time-Dependent Factors Associated with Survival among 1,138 Persons First Prescribed Any Triple Combination Antiretroviral Therapy

The same analyses were repeated with non-accidental deaths as the outcome of interest (*n =* 160; 77.3%). Individuals dying from non-accidental deaths in the study had less experienced physicians (median: 16 patients per physician [inter-quartile range: 2, 81 patients per physician]), worse CD4 cell counts (median: 150 cells/mm^3^ [inter-quartile range: 45, 310 cells/mm^3^]), and higher viral load levels (median: 175,000 copies/ml [inter-quartile range: 88,000, 405,000 copies/ml]) than the individuals included in the all cause mortality (see Table 1). When we assessed the association between survival and resistance in this population we observed that persons who exhibited reduced sensitivity to any antiretroviral had death rates that were 1.51 times (95% CI: 1.04, 2.20) higher than those who did not exhibit resistance. We also looked at the individuals dying via accidental death (*n =* 47; 22.7%). These individuals had more experienced physicians (median: 53 patients per physician [inter-quartile range: 12, 131 patients per physician]), better CD4 cell counts (median: 300 cells/mm^3^ [inter-quartile range: 210, 470 cells/mm^3^]), and lower viral load levels (median: 129,000 copies/ml [inter-quartile range: 39,000, 210,000 copies/ml]) than the individuals included in the all cause mortality (see Table 1). When we assessed the association between survival and emergence of resistance in this population we observed that the hazard ratio was 1.83 (95% CI: 0.92, 3.63).

Table 4 presents the final marginal structural models of the causal effect of drug resistance on mortality. In all models, the time-dependent confounders were protease inhibitor/non-nucleoside experience, AIDS diagnosis, CD4 cell count, viral load, and HAART regimen. The exposures of main interest were emergence of any resistance, and emergence of resistance to each separate class of mutation (lamivudine, other nucleosides, non-nucleosides, and protease inhibitors). Model 1 considers the emergence of any resistance as the exposure of main interest. The results show that persons who developed any resistance had death rates that were 1.75 times (95% CI: 1.27, 2.43) higher than those who did not exhibit any resistance. When we considered each separate class of mutation as the exposure of interest (Models 2–5), while controlling for the presence/absence of other mutations, we observed that those persons who exhibited reduced sensitivity to non-nucleoside reverse transcriptase inhibitors had the highest risk: they were 3.02 times (95% CI: 1.99, 4.57) more likely to die than those who did not exhibit this type of resistance.

**Table 4:**
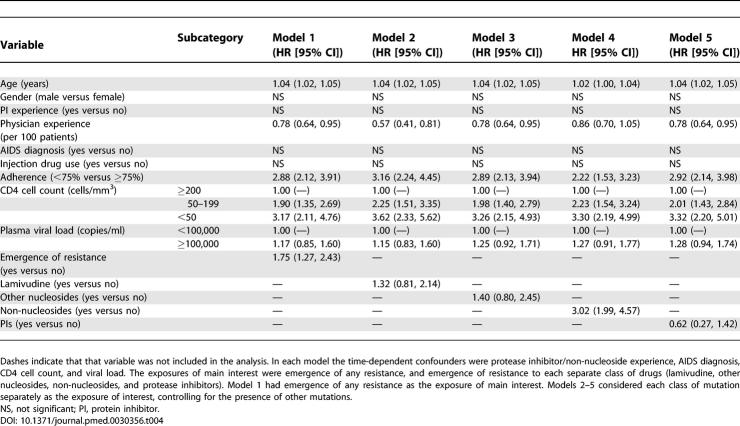
Hazard Ratio Estimates from the Marginal Structural Model of the Causal Effect of Drug Resistance on Mortality

## Discussion

Our results demonstrate that emergence of resistance to non-nucleoside reverse transcriptase inhibitors was associated with a greater risk of subsequent death than was emergence of resistance to any other class of drug. Our finding that resistance to non-nucleoside reverse transcriptase inhibitors was significantly associated with mortality even after adjustment for plasma HIV-1 RNA levels, adherence, and CD4 cell counts suggests resistant viruses may have differences in fitness [19]. Resistance to non-nucleoside reverse transcriptase inhibitor therapy has also previously been shown to occur at low to moderate levels of adherence, indicating that non-nucleoside reverse transcriptase resistance may be an independent marker for poor adherence in our population [20]. Mortality was not associated with viruses resistant to protease inhibitors, lamivudine, or other nucleoside reverse transcriptase inhibitors.

The emergence of drug resistance to antiretrovirals in this drug-naïve cohort remains low [8]. The median time to first emergence of resistance to any class of drug was just over a year. HIV-drug resistance to any class was observed in just over a quarter of participants over 30 mo, with less than 10% of patients exhibiting resistance to all three classes. In contrast to other studies that included both therapy-naïve and experienced patients [6], our results are likely much closer to true levels of multiple drug resistance in a population initiating HAART.

Most patients in our study died with wild-type virus. Nearly, three-quarters of the deaths were of patients who exhibited no resistance to any class of drug, and less than 2% exhibited resistance to all three classes. Such patients may have had insufficient drug exposure because of poor adherence, thus selecting for resistant virus. We have previously shown significant differences in survival among adherent and non-adherent patients at all levels of CD4 count, with a particularly dramatic impact on survival in patients with highly advanced disease [9]. These findings are independent of whether the measurement of adherence is based on prescription refills or antiretroviral concentrations in untimed plasma samples [9,21–23]. Similarly, in an indigent cohort in San Francisco, the level of adherence was closely associated with longitudinal viral suppression and the rate of subsequent disease progression [24].

Two recent studies have reported on the association between drug resistance and risk of death [8,25]. Lucas et al. [25], based on a cohort of non-naïve individuals starting HAART, found that emergence of resistance was not linked to increased mortality rates in a multivariable analysis, adjusting for prognostic factors such as HIV-1 exposure group, CD4 cell count at resistance testing, mean log_10_ HIV-1 RNA level during prior treatment with HAART, and whether or not HAART was used during the 6 mo following resistance testing. There are two important differences between this study and ours. First, Lucas et al. failed to control for several other important fixed and time-varying confounder factors that could have influenced their results, such as adherence, HAART regimen, and injection drug use, to cite a few. Second, they did not exclude from the analysis individuals who had received HAART previously, and who had already presented some type of mutation, which could have potentially biased their results. Another study, by Recsky et al. [8], based on a subpopulation of individuals from our study that died between July 1997 and December 2001, also failed to demonstrate an association between drug resistance and mortality. The authors of this latter paper used univariable analyses to compare the presence or absence of resistance at the time of death across several characteristics: age (at initiation of HAART and at death), sex, injection drug use status, CD4 cell count and plasma HIV-1 RNA level at the time right before the genotype test, adherence, and time undergoing treatment. The only difference they found between these two groups was due to the duration of treatment, where individuals who presented any drug resistance had longer median duration of therapy than those without any resistance (32 versus 15 mo; *p* < 0.001). We believe that the differences among the results of these two studies and ours are due to differences between study populations (e.g., comparison groups and definitions of cohort) and differences in methodology used to assess the relationship between outcome and exposures.

Although we concluded that emergence of resistance was strongly associated with elevated risk of mortality, there are two important potential sources of bias that could explain our results. First, HOMER patients with samples of HIV-1 RNA of less than 1,000 copies/ml during the entire follow-up period (*n =* 229; 20.1%) were assumed to have no drug-resistance mutations. We therefore conducted a sensitivity analysis restricted to patients with plasma viral load levels of 1,000 copies/ml or more to assess the impact of this assumption on our results and the robustness of the marginal structural models in dealing with this possible source of bias. We observed that patients who showed the emergence of any resistance were 1.68 times (95%CI: 1.19, 2.38) more likely to die than those who did not, which is consistent with the results found in our original analysis. Second, in the present study we did not exclude patients who achieved durable viral elevation (HIV-1 RNA levels ≥ 1,000 copies/ml) during the entire follow-up period (*n =* 130; 11.4%). To assess whether this group drove our mortality trends, and not emergence of resistance per se, we conducted a second sensitivity analysis restricted to patients with no durable viral elevation. We observed that those patients that developed any type of resistance were 2.09 times (95%CI: 1.41, 3.10) more likely to die than those who did not, which is also consistent with our previous results.

There are several features of our study that should be highlighted. First, our study was carried out within a province-wide treatment program, in which all individuals had access to medical attention, combination antiretroviral therapy, and laboratory monitoring free of charge. We are confident, therefore, that our results are not influenced by access to therapy, a factor that has often compromised the interpretation of similar population- and cohort-based studies. Second, this study was based on treatment-naïve individuals, thus our results are not confounded by previous therapy use. Third, delayed reporting was not likely a factor; as the vast majority of deaths are reported within 3 mo of death through active follow-up with physicians and hospitals and regular linkages. Finally, although we adjusted our analyses for pertinent demographic and clinical characteristics, like all studies of patients treated in observational cohorts, unmeasured differences may exist among study populations, and for this reason caution is warranted.

In summary, we demonstrated that emergence of resistance to non-nucleoside reverse transcriptase inhibitors was associated with a greater risk of subsequent death than was emergence of resistance to other drug classes. Future research is needed to identify the particular subpopulations of men and women at greatest risk. These efforts also need to elucidate the impact of resistance over a longer follow-up period.

## Supporting Information

### Accession Numbers

The GenBank (http://www.ncbi.nlm.nih.gov/Genbank) accession number for HIV HXB2 is K03455.

## Abbreviations

CI: confidence interval
Centre: British Columbia Centre for Excellence in HIV/AIDS
HAART: highly active antiretroviral therapy
HOMER: HAART Observational Medical Evaluation and Research
